# Identification of potential mutations and genomic alterations in the epithelial and spindle cell components of biphasic synovial sarcomas using a human exome SNP chip

**DOI:** 10.1186/s12920-015-0144-7

**Published:** 2015-10-27

**Authors:** Yan Qi, Ning Wang, Li-Juan Pang, Hong Zou, Jian-Ming Hu, Jin Zhao, Jun Zhang, Chun-Xia Liu, Wen-Jie Zhang, Xiang-Lin Yuan, Feng Li

**Affiliations:** Department of Oncology, Tongji Hospital, Tongji Medical College of Huazhong University of Science and Technology, Wuhan, Hubei 430000 China; Department of Pathology, Shihezi University School of Medicine, North 4th Road, Shihezi, 832002 Xinjiang China; Key Laboratories for Xinjiang Endemic and Ethnic Diseases, Shihezi University and Chinese Ministry of Education, Shihezi, Xinjiang 832002 China; Department of Medical Genetics, Shihezi University School of Medicine, Bei-Er-Lu, Shihezi, Xinjiang 832000 China

**Keywords:** Biphasic synovial sarcomas, Genome-wide SNP analysis, Epithelial and mesenchymal components

## Abstract

**Background:**

Synovial sarcoma (SS) is one of the most aggressive soft-tissue sarcomas and is noted for late local recurrence and metastasis. It is of uncertain histological origin and exhibits a biphasic histopathological form involving both the mesenchyme and epithelium. Thus, its diagnosis and therapy remain a huge challenge for clinicians and pathologists. This study aimed to determine whether differential morphological-associated genomic changes could aid in ascertaining the histogenesis of SS and to determine whether these sarcomas showed some specific mutated genes between epithelial and spindle cells that would promote tumor invasion and metastasis.

**Methods:**

We conducted a comprehensive genomic analysis of mesenchymal and epithelial components in 12 formalin-fixed paraffin-embedded biphasic SS samples using the Illumina human exon microarray. Exome capture sequencing was performed to validate the single nucleotide polymorphism (SNP)-chip data, and *de novo* data were generated using a whole-exome chip with the Illumina exon microarray. Fisher’s exact test based on PLINK analysis of the SNP-chip data.

**Results:**

Here, the SNP-chip data showed that 336 SNPs had association *P*-values of less than 0.05 by chi-square test. We identified 23 significantly mutated genes between epithelial and spindle cell regions of SSs. Fifteen gene mutations were specific for the spindle cell component (65.2 %) and eight for the epithelial cell component (34.8 %). Most of these genes have not been previously reported in SS, and neuroguidin (*NGDN*), RAS protein activator like 3 (*RASAL3*), *KLHL34* and *MUM1L1* have not previously been linked to cancer; only one gene (*EP300*) has been reported in SS. Genomic analyses suggested that the differential SNPs in genes used for functional enrichment are mainly related to the inflammatory response pathway, adhesion, ECM–receptor interactions, TGF-β signaling, JAK–STAT signaling, phenylalanine metabolism, the intrinsic pathway and formation of fibrin.

**Conclusions:**

This study investigated novel biological markers and tumorigenic pathways that would greatly improve therapeutic strategies for SS. The identified pathways may be closely correlated with the pathogenic mechanisms underlying SS, and SS development is associated with morphological features.

**Electronic supplementary material:**

The online version of this article (doi:10.1186/s12920-015-0144-7) contains supplementary material, which is available to authorized users.

## Background

Synovial sarcoma (SS) is a highly aggressive mesenchymal tumor with typical dual epithelial and mesenchymal differentiation [1]. It affects pediatric, adolescent, and adult populations and comprises approximately 10 % of all soft-tissue sarcomas; the age-standardized incidence rate per million individuals ranges from 0.5 to 1.3 [2]. SS has a wide spectrum that includes two main variants: monophasic fibrous SS, the most common variant and biphasic SS (BSS), which displays glandular epithelial differentiation architecture in a background of spindle cells. SS likely originates from undifferentiated mesenchymal tissue with variable epithelial differentiation and highly specific chromosomal translocation in more than 95 % of cases [3]. However, the molecular mechanisms underlying tumorigenesis and dual differentiation have remained elusive.

Much of the available literature on SS comprises case reports. Research on the pathogenesis of SS is mainly focused on the relationship between fusion genes and diagnosis or prognosis. However, the cell-of-origin, dual differentiation of SS and the histological transitional relationships between epithelial and mesenchymal differentiation are unclear.

Recent advances in genomic technologies have offered a great opportunity for identifying the complete biological characteristics of neoplastic tissues, resulting in improved diagnosis, treatment selection, rational classification based on molecular carcinogenesis, and identification of therapeutic targets. Genome-wide single nucleotide polymorphism (SNP) analysis is a powerful approach that allows for the investigation of genomes and transcriptomes using limited sample material, and identification of disease-causing genes; other advantages include its abundance in the human genome and ease of high-throughput typing therapy [4]. Thus, these methods have great potential for guiding therapy.

Because of the relative rarity of SS and the poor prognosis, it is not easy to collect an adequate number of SS samples for a genome-wide association study. However, new cost-effective technologies such as microarray SNP-chip typing and whole exome sequencing have allowed us to identify candidate genes, mutations and potential targets for further study and validation [5]. Here, we integrated the resulting data for 319 target genes with those from existing open-source resources with the following aims: detection of somatic variants between the epithelial and mesenchymal components in SS by genome-wide SNP analysis; linkage of target genes with additional biologically significant pathways and gene sets; and to provide insights into our previous mechanistic studies, thereby providing the basis for further experiments for characterizing the genetic factors that are involved in pathogenesis and that could aid in future targeted therapies for SS.

## Methods

### Patients and tissue specimens

Twelve formalin-fixed, paraffin-embedded (FFPE) BSS samples that were confirmed to have a fusion gene and obtained from patients treated at the Department of Pathology, First Affiliated Hospital, Shihezi University, School of Medicine, between 1980 and 2011, were included in this series. Clinical and demographic data were obtained from the medical charts. The diagnosis of SS was confirmed by histological and immunohistochemical analyses and the presence of a fusion gene was detected by One Step RT-PCR (QIAGEN, Venlo, The Netherlands). The characteristics of the 12 BSS patients enrolled in this study are shown in Table 1. Clinical staging was performed according to the National Comprehensive Cancer Network 2012 guidelines for soft-tissue tumors. Follow-up surveys were conducted.

**Table 1:** Clinical Description of BSSs Patients for Genome-Wide SNP

Case	sex/age	Location	Size (cm)	FNCLCC	pTNM	Metastases	Status at last follow-up	Fusion gene
1	F/55	left ilium	5	3	IV	lung	DOD	SYT-SSX1
2	F/22	Left heel	3	3	IIA		NA	SYT-SSX1
3	M/40	Right groin	22	2	IV	liver	AWD 9 months after presentation	SYT-SSX2
4	F/10	Right elbow	5	3	IV	Bone marrow cavity	DOD	SYT-SSX1
5	M/64	Left hip	11.5	3	IV	lung	DOD	SYT-SSX1
6	M/32	Oral	3	3	I	lung	DOD	SYT-SSX1
7	F/37	Right tibia	6; 5	3	III		DOD	SYT-SSX1
8	F/21	Right thigh	7.5	3	I		AWD 27 months after presentation	SYT-SSX1
9	M/55	Right foot	5.2	3	I	lung	DOD	SYT-SSX1
10	M/47	Left leg	5	3	IV	lung	DOD	SYT-SSX2
11	M/39	Left hand and forearm	6	3	IV	Bone	DOD	NA
12	M/52	Right kidney	5	3	III		AWD 23 months after presentation	SYT-SSX2

*F* female, *M* male, *NA* not available, *BSS* biphasic synovial sarcoma, *AWD* alive with disease, *DOD* died of disease, *NA* not available

Written informed consent was obtained from all patients regarding the use of the collected samples in research studies. The patient records and information were anonymized and de-identified before analysis. Human subjects in this study provided informed consent for use of their tissues for research purposes following procedures approved by the Clinical Research Ethics board of the First Affiliated Hospital, Shihezi University School of Medicine.

### Tissue preparation and microscopic separation of the epithelial and spindle cell components of BSSs

Laser capture microdissection (LCM) and tissue chips were used for separation of the epithelial and spindle cell components in12 cases of biphasic SS. For LCM, paraffin sections were deparaffinized in xylene, rehydrated through a graded alcohol series to distilled water, and then stained with hematoxylin. Epithelial cells and spindle cells were isolated from the slides using LCM (PixCell II Laser Capture Microdissection System; Arcturus Engineering Inc. Mountain View, CA, USA). The cells were captured using a 30-μm pulse to focally melt a thermoplastic membrane attached to a transparent flat cap (PixCell II Laser Capture Microdissection System). After LCM, the cap containing the captured tissue was placed on a 0.5-mL standard Eppendorf microfuge tube.

For tissue chip separation, regions of the epithelial cell and spindle cell components in the paraffin block were selected by comparing the results of hematoxylin eosin staining. A hollow needle (diameter, 1.0 mm) was used to puncture the selected area to a new small wax block.

### DNA extraction and SNP array

Genomic DNA was isolated from the FFPE tumor samples using the QIAamp DNA Micro Kit (Qiagen Inc., Valencia, CA, USA) in accordance with the manufacturer’s instructions. The lowest amount of genomic DNA for which the genomic SNP chip analysis was successful was 1 μg. The DNA was extracted from 24 FFPE tissue samples (12 patients), with quality metrics of A260/280 = 1.7–2.0 and A260/230 > 1.6. DNA targets from patients were prepared and hybridized to Infinium HD Assay Super chips following the manufacturer’s recommendations. Quality analysis was performed by importing the CEL files into Illumina Human Exome-12v1.1 according to the “Quality Control Assessment in Genotyping Console”. Association analysis was performed for control versus case with the PLINK (http://pngu.mgh.harvard.edu/~purcell/plink) software, using the Fisher’s exact test, chi-square test and estimated odds ratio. Pearson chi-square values and *P*-values (chi-square and Fisher’s exact test) were calculated using Haploview 4.2. PLINK data.

### Illumina exon microarray detection

A custom microarray with exon level resolution was developed to identify gross deletions and duplications. Study samples were processed using Illumina Human Exome-12v1.1 (Beijing Compass Biological Technology Co. Ltd; http://www.kangpusen.com/index.html). The microarray contains approximately 60,000 integrated oligonucleotide probes that have been annotated against the human genome assembly build. The probes’ density was increased in the exons and 300 bp flanking intronic sequence. In addition, probes were placed at every 2.5 Kb of the intronic sequence and heavily tiled in promoter regions. After validation runs, only probes with optimal performance were selected for the final array design.

Briefly, the standard DNA plate was prepared first, and then the standard QNT plate with diluted PicoGreen and the sample QNT plate with PicoGreen and DNA were prepared. Subsequently, the DNA samples were shifted to the MSA1 plate. The samples were denatured and neutralized, and then prepared for amplification by overnight incubation. The next day, the DNA was enzymatically fragmented using end-point fragmentation to avoid over-fragmentation. Then, the DNA samples were treated with 2-propanol and PM1 to precipitate MSA1, and the precipitated DNA was resuspended using RA1. Next, the fragmented resuspended DNA samples were dispensed on BeadChips. The BeadChips were incubated in the Illumina hybridization oven to hybridize the samples onto the BeadChips. The incubation time was appoximately16–24 h. The next day, the BeadChips were prepared for the staining process, and the unhybridized and non-specifically hybridized DNA was washed off. Labeled nucleotides were added to extend the primers hybridized to the DNA. The primers were stained, the flow-through chambers were disassembled, and the BeadChips were coated for protection. The iScan System and the Illumina BeadArray Reader were using by the Illumina GenomeStudio Genotyping Module. There are eight categories of internal control in an exon array Illumina SNP microarray experiment for each sample: staining, extension, hybridization, stringency, target removal, restoration, non-specific binding and non-polymorphic controls. Quality analysis of the Illumina HumanExome-12v1.1 report samples showed that the call rate was 0.7526.

### Functional annotation

WebGestalt (WEB-based GEne SeT AnaLysis Toolkit; http://bioinfo.vanderbilt.edu/webgestalt) [6], which involves the KEGG database, Pathway Commons and WikiPathways Analysis, was used for functional enrichment analysis and hierarchical clustering of SNPs detected by SNP Chip. *P*-values were calculated at medium classification stringency using a modified Fisher’s exact test (EASE score), and terms with *P*-values of < 0.05 were considered significantly enriched. The enriched protein interaction network modules were visualized using String network online analysis (http://www.string-db.org/newstring_cgi/show_input_page.pl?UserId=nXWzWNEgLwwF&sessionId=Gfs9PMrT96ZB).

## Results

### Patients’ characteristics

The study cohort included 12 patients with definite epithelial and spindle cells in BSSs. The patients were predominantly male (seven [58.3 %] versus five female patients [41.7 %]), with the age at diagnosis ranging from 10 months to 64 years (mean age, 39 years). The tumors had a wide anatomical distribution; however, most arose in the upper and lower limbs (6, 38 %), and the head and neck region (5, 31 %). A total of five tumors (25 %) occurred in the retroperitoneum, hip, spinal canal, lung and thoracic wall. Clinical follow-up was available for 11 cases. The mortality rate was very high, that is, 63.6 % (7 of 11 patients) patients died of the disease or of subsequent metastasis. Some of the surviving patients experienced local recurrence or liver and lung metastases. The data are listed in Table 1.

### Exome-chip analysis

**Fig. 1:**
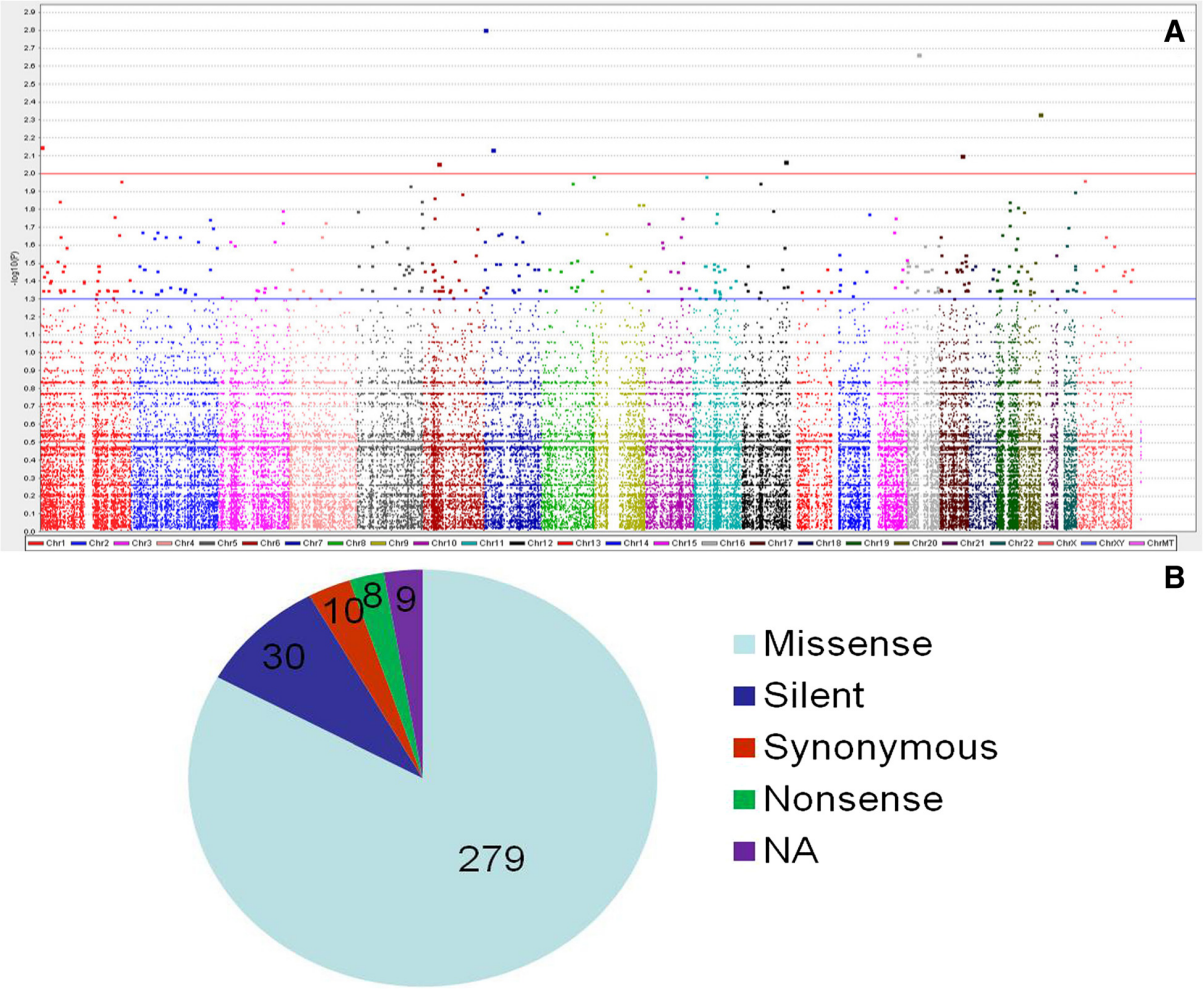
Genome-wide analysis of a total of 2,345,678 SNPs in epithelial and spindle components in 12 cases of biphasic synovial sarcomas. **a** Manhattan plot showing negative log-transformed *P*-values of the case–control allele frequency significance on the y-axis. The color scale of the x-axis denotes chromosome numbers. Gene names associated with individual dots indicate SNPs of greatest significance or with potential disease relevance. **b** Mutation types of 343 SNPs meeting the established criterion for genome-wide significance (*P* < 0.05, chi square test)

We performed exome capture sequencing to validate the SNP chip data and to identify SNPs not present on the array. Approximately 249,101 single-nucleotide variants and small insertion and deletion changes were discovered for the epithelial and spindle cells components in the genomes of the 12 patients on comparison with the current reference haploid human genome sequence (Fig. 1a). The Fisher’s exact test based on PLINK analysis of the SNP-chip data showed that 336 SNPs had association *P*-values of less than 0.05 (*P* < 0.05, chi square test) Additional file 1. Because our studies were limited by the small number of patients with BSSs, we set an initial threshold for genome-wide significance of less than 10–3 by the chi-square test and further analyzed variants meeting this criterion. Analysis with the PLINK and Haploview software showed that a total of 23 SNPs in 23 genes (*CTH*, *RBM44*, *LARS*, *HNRNPA*, *ADAMTS2*, *F13A*, *TNXB*, *ADAP1*, *PIK3CG*, *GCN1L1*, *NGDN*, *AHNAK2*, *ACAN*, *PRR14*, *DSEL*, *ETV2*, *DERL3*, *CELSR1* and *BCORL1*) had association *P*-values of less than 10–3 (Fig. 1b). All these SNPs were located in exonic regions. Mutations in 15 genes were specific for the spindle cell component (15/23, 65.2 %) (*CTH*, *RBM44*, *LARS*, *HNRNPA*, *F13A*, *PIK3CG*, *GCN1L1*, *NGDN*, *AHNAK2*, *PRR14*, *RASAL3*, *DERL3*, *CELSR1*, *KLHL34* and *EP300*), and those in eight genes were specific for the epithelial cell component (8/23, 34.8 %) (*ADAMTS2*, *TNXB*, *ADAP1*, *ACAN*, *DSEL*, *ETV2*, *MUM1L* and *BCORL1*).

The most highly ranked SNPs associated with genes are presented in Table 2. We identified 23 significantly mutated genes between the epithelial and spindle cell areas of SS. Most of these genes have not been previously reported in SSs, and four genes, neuroguidin (NGDN), RAS protein activator like 3 (RASAL3), *KLHL34* and MUM1L1, have not been previously linked to cancer. Only one gene (*EP300*) has been reported in SS. *NGDN* is an EIF4E (MIM 133440)-binding protein that interacts with CPEB and functions as a translational regulatory protein during the development of the vertebrate nervous system [7]. *RASAL3*, a member of the RAS signaling pathway, is located at 19p13.12 and contains 18 exons; it was identified in 2004 [8]. *KLHL34* is a member of the Kelch-like (KLHL) gene family, which encodes a group of proteins that generally possess a BTB/POZ domain, a BACK domain and 5–6 Kelch motifs. *KLHL* genes are responsible for several Mendelian diseases and have been associated with cancer. Further investigation of this family of proteins will likely provide valuable insights into basic biology and human disease [9]. The melanoma-associated antigen (mutated) 1-like 1 gene (*MUM1L1*) has four transcripts (splice variants). This gene encodes a protein that contains a mutated melanoma-associated antigen 1 domain. Proteins that contain mutated antigens are probably expressed at high levels in certain types of cancers. Multiple alternatively-spliced variants, encoding the same protein, have been identified. *EP300* somatic mutations were significantly different between the epithelial and spindle cell components, and were mainly located in the DNA-binding domain and the mutated gene encoding histone H3 lysine acetyltransferases. EP300 is a very important factor in the TGF-β signaling pathway. SNPs located in genes with unknown functions or without obvious importance to disease pathogenesis are not discussed here.

**Table 2:** Highly ranked SNPs associated with BSS

SNPs Name	Chr	MapInfo	Alleles	Location	*P* value^a^	Gene(s)	Mutation(s)	Bp	F_C
exm68141	1	70890007	[A/G]	EXON	0.00904	CTH	Missense_V166M, V134M,Silent	70890007	0.222
exm279897	2	238742929	[T/C]	EXON	0.00904	RBM44	Missense_I1015T	238742929	0.222
exm2090870	5	145537108	[T/C]	EXON	0.007661	LARS	Missense_R308H	145537108	0.625
exm508367	5	177633872	[A/T]	EXON	0.004678	HNRNPAB	Missense_Y173F,	177633872	1
exm509539	5	178634672	[T/C]	EXON	0.008741	ADAMTS2	Missense_V245I	178634672	0.583
exm514146	6	6167833	[A/T]	EXON	0.003445	F13A1	Missense_L589Q	6167833	1
exm534246	6	32038079	[T/G]	EXON	0.00572	TNXB	Missense_F1701L	32038079	0.357
exm597614	7	975046	[C/G]	EXON	0.001565	ADAP1	Missense_V60L	975046	1
exm648916	7	106515241	[T/C]	EXON	0.007888	PIK3CG	Missense_A795V	106515241	0.3
exm1042751	12	120580489	[T/C]	EXON	0.00394	GCN1L1	Missense_R1884Q	120580489	0.333
exm1091040	14	23945331	[C/G]	EXON	0.006545	NGDN	Missense_V172L	23945331	0.444
exm1133291	14	105415882	[T/C]	EXON	0.003676	AHNAK2	Missense_R1969Q	105415882	0.5
exm1186826	15	89401134	[T/C]	EXON	0.008741	ACAN	Missense_L1773P	89401134	0.5
exm1233625	16	30666358	[A/G]	EXON	0.004862	PRR14	Missense_R356Q	30666358	0.556
exm1391659	18	65180127	[A/C]	EXON	0.007982	DSEL	Missense_L583F	65180127	0.5
exm1925094	19	15567000	[A/G]	EXON	0.004898	RASAL3	Missense_C546R	15567000	0.313
exm1456897	19	36134208	[A/G]	EXON	0.005775	ETV2	Missense_D90N	36134208	0.438
exm1593372	22	24179922	[G/C]	EXON	0.006903	DERL3	Missense_F149L	24179922	0.389
exm1618561	22	46763654	[T/C]	EXON	0.003973	CELSR1	Missense_Y2684C	46763654	0.444
exm1631314	X	21674603	[A/G]	EXON	0.006928	KLHL34	Missense_A435V	21674603	0.667
exm1651515	X	105451287	[T/A]	EXON	0.009534	MUM1L1	Missense_D621V	105451287	1
exm1656996	X	129149891	[A/G]	EXON	0.004388	BCORL1	Missense_R1048Q	129149891	0.25
exm1611652	22	41546158	[A/C]	EXON	0.004417	EP300	Missense_S106G	41546158	0.611

^a^Chi-square test; F_C, the frequency of this variant in cases

### Functional annotation of SS-associated variants

**Fig. 2:**
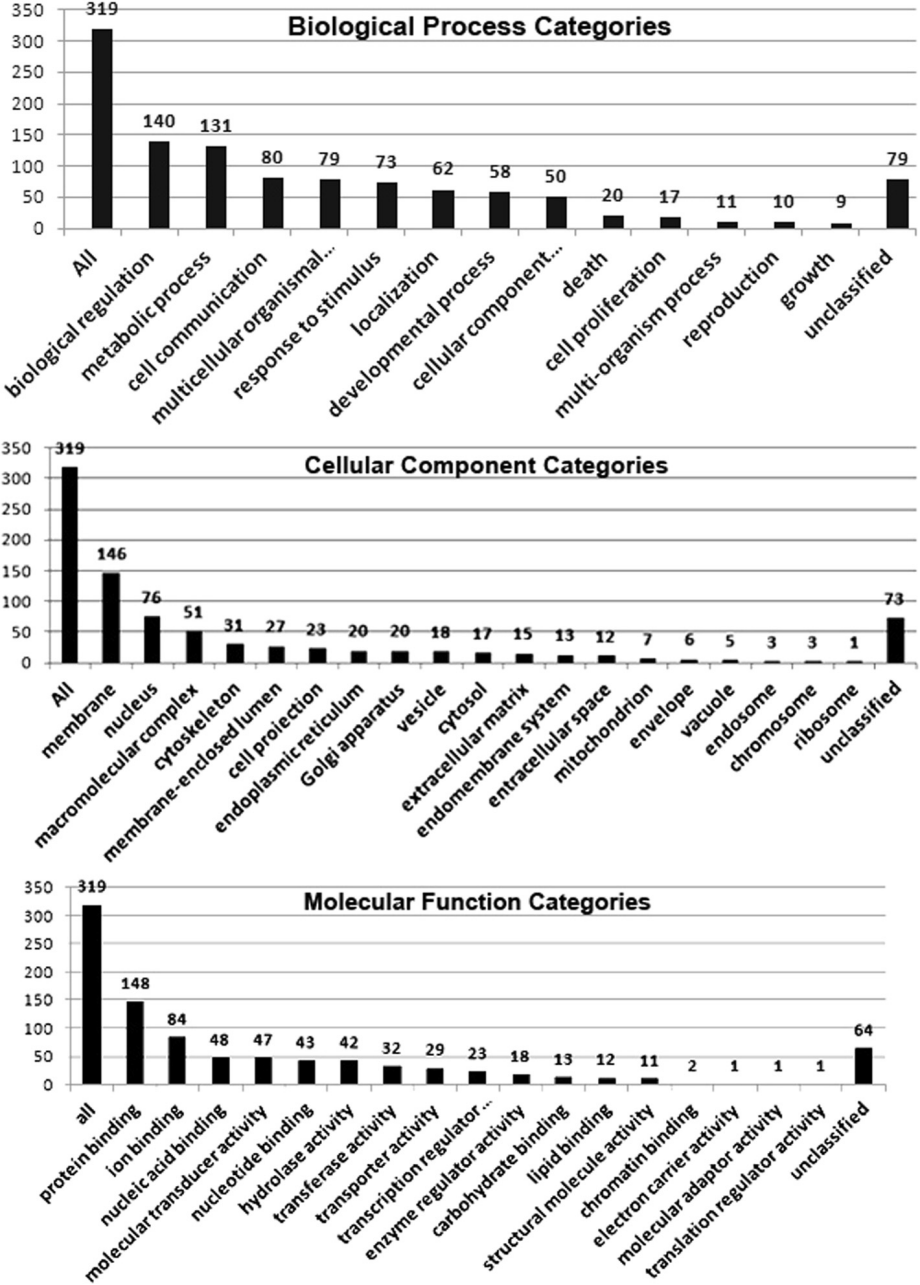
GO output enrichment analysis of differential SNPs for target genes. Distribution of significant genes in gene function (molecular function) and cell components (cellular component) categories, and those that participate in biological processes (in process)

**Fig. 3:**
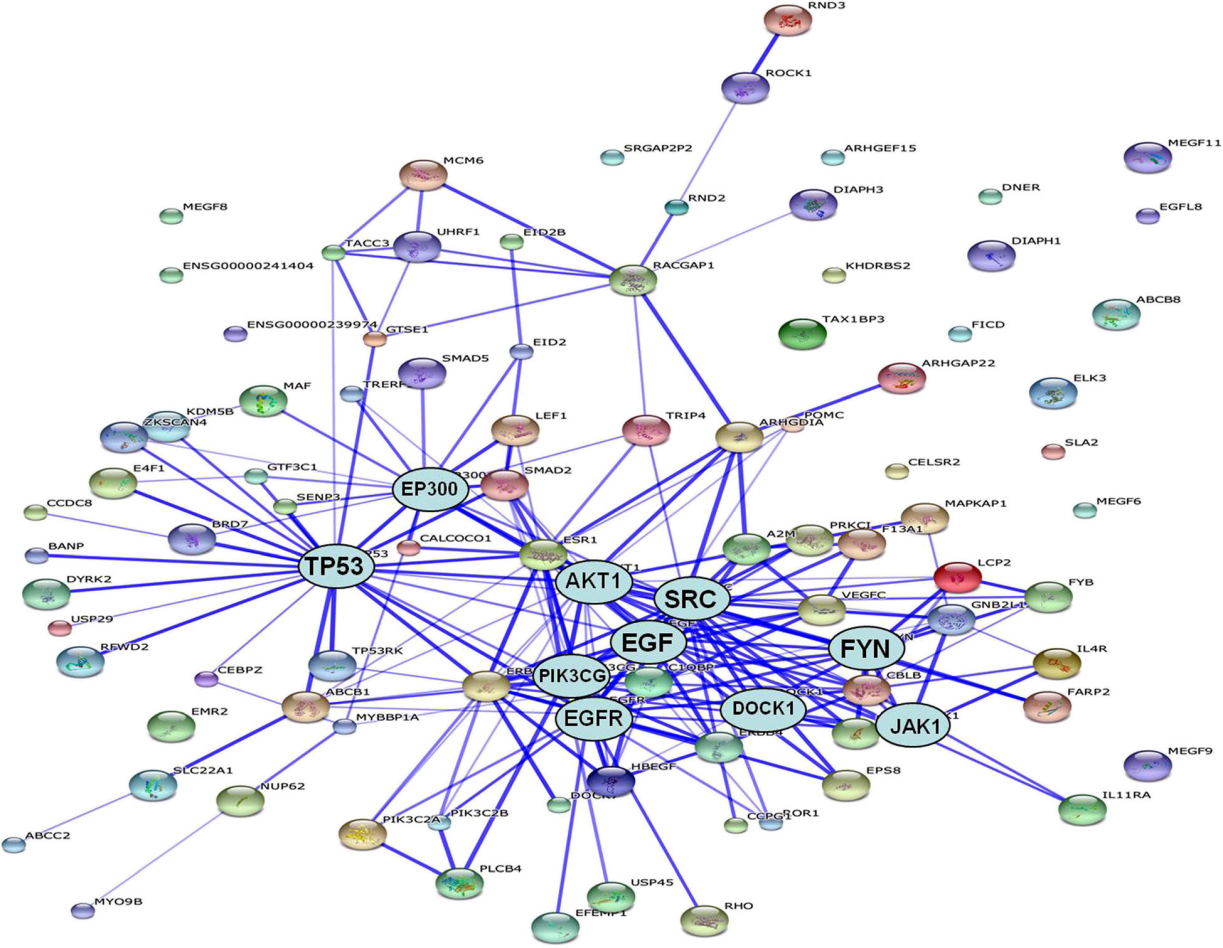
STRING network analysis reveals a database of known and predicted protein interactions. The interactions include direct (physical) and indirect (functional) associations. The network has been simplified for clear illustration of genes of interest. Stronger associations are represented by thicker lines. The significant genes were mainly *TP53*, *AKT1*, *SRC*, *EGF*, *EGFR*, *PIK3CG*, *EP300* and *FYN*, and enriched functional categories included focal adhesion, cytokine–cytokine receptor interactions, the ERBB signaling pathway, the cell cycle, adherens junctions and the JAK–STAT signaling pathway

We analyzed 319 genes containing 336 SNPs significantly associated with BSSs by SNP-chip analysis using the WebGestalt functional clustering algorithm (Table 3). GO analysis showed three aspects: biological processes (biological regulation [43.9 %, 140/319] and metabolic processes [41.1 %, 131/319]); cellular components (membrane [45.8 %, 146/319] and nucleus [23.8 %, 76/319]); molecular function (protein binding [46.4 %, 148/319] and ion binding [26.3 %, 84/319]) (Fig. 2). SNPs were detected in genes with potential relevance for epithelial and spindle cell components of BSSs by Commons Pathway Additional file 2 and Functional Enrichment Analysis (WikiPathways Additional file 3 and KEGG pathways Additional file 4), mainly in genes such as those involved in the inflammatory response pathway, focal adhesion, the TGF-β signaling pathway, ECM–receptor interactions, phenylalanine metabolism and the JAK–STAT signaling pathway (Table 3). STRING network online analysis was used for additional evaluation of the enriched protein interaction network. The results showed enrichment of functional categories including focal adhesion, cytokine–cytokine receptor interactions, the JAK–STAT signaling pathway, the ERB signaling pathway, the cell cycle, adherens junctions and the Wnt signaling pathway (Fig. 3).

**Table 3:** Functional enrichment analysis of top ranked SNPs

Different riched methods	Related pathways of riched functional analysis	*P* value	Related genes of riched functional analysis
Wikipathways pathway	Inflammatory Response Pathway	0.0013	IL4R, LAMC2, LAMA5, COL1A1
	Focal Adhesion	0.0029	SPP1, COL1A1, COL5A1, EGF, TNXB, ROCK1, LAMC2, PIK3CG, LAMA5
	TGF Beta Signaling Pathway	0.0097	EP300, SPP1, JAK1, EGF
KEGG pathway	ECM-receptor interaction	0.0004	GP9, SPP1, LAMA5, LAMC2, COL1A1, COL5A1, TNXB
	Focal adhesion	0.0015	SPP1, FLT4, COL1A1, COL5A1, EGF, TNXB, ROCK1, LAMC2, LAMA5, PIK3CG
	Phenylalanine metabolism	0.0044	IL4I1, AOC2, ALDH3B2
	Jak-STAT signaling pathway	0.0063	IFNE, EP300, IL4R, IL11RA, JAK1, PIK3CG, CBLB
	Hematopoietic cell lineage	0.0085	CD1E, GP9, IL4R, IL11RA, CD22
	Inositol phosphate metabolism	0.0111	PLCB4, PIK3C2B, PIK3CG, PIK3C2A
Pathway Commons pathway	Intrinsic Pathway	0.0015	GP9, C1QBP, A2M, F13A1
	Formation of Fibrin Clot (Clotting Cascade)-	0.0021	GP9, C1QBP, A2M, F13A1
	Hemostasis	0.0054	TTN, OL1A1, A2M, EGF, F13A1, GP9, MAG, C1QBP
	Platelet Activation	0.0054	GP9, COL1A1, A2M, EGF, F13A1
	Exocytosis of Alpha granule	0.0050	GP9, A2M, EGF, F13A1
	Platelet degranulation	0.0059	GP9, A2M, EGF, F13A1
	Terminal pathway of complement	0.0037	C8G, C7
	vWF interaction with collagen	0.0037	GP9, COL1A1

## Discussion

SSs harbor a chromosomal translocation t(X; 18)(p11.2; q11.2), which produces the SS-specific fusion gene SYT–SSX. Although the precise function of SYT–SSX has not yet been studied, accumulating evidence suggests its role in gene regulation via epigenetic mechanisms, and the product of SYT–SSX target genes may serve as biomarkers of SS [10, 11]. However, lack of knowledge regarding the cell-of-origin of SS has hampered identification of its targets [12]. The clinical outcomes of SS are very poor and approximately 90 % of patients die because of metastasis. Understanding the process by which tumor cells destroy the basement membrane, invade and metastasize is essential for the advancement of SS treatment strategies and improvement of survival. An intriguing observation in SS is that the specific gene fusion (*SYT*–*SSX1* vs. *SYT*–*SSX2*) correlates strongly with the tumor phenotype (monophasic vs. biphasic histology, as defined by the presence of glandular epithelial differentiation with lumen formation), and almost all BSS has been shown to harbor the *SYT*–*SSX1* fusion gene [13, 14]. Our results showed that most cases are *SYT*–*SSX1* fusion genes (8/11). Moreover, *SYT*–*SSX* fusion transcripts are found in both epithelial and spindle cell areas of BSS [15].

The recent advances in high-throughput sequencing technologies are rapidly becoming common practice. A large amount of data are generated using these methods and are becoming an important resource for deciphering the genotype underlying a given phenotype in clinical diagnosis, cancer research and molecular targeted therapies for various cancers [4, 16]. Because of the relative rarity of SS and its poor prognosis, it is not easy to collect adequate numbers of fresh samples of SS for a genome-wide association study. Therefore, it is very difficult to analyze subtle differences in pathogenesis and clinical benefits on the basis of the histological type. Therefore, future more in-depth studies should consider these issues in the study design when FFPE tissue samples are used. Samples stored in diagnostic pathology archives represent an invaluable biobank for retrospective clinical and molecular research. This is especially true in the case of SS, which is a rare malignant tumor, and FFPE tissue samples can alleviate or eliminate the need for the tedious collection and storage of cryopreserved clinical samples. It is more difficult to extract nucleic acids from FFPE tissue because of the need to remove the paraffin and to counteract covalent protein–DNA interactions that result from the fixation process [17]; however, Schweiger et al. [18], who first reported NGS-based analysis of DNA (DNA-Seq), had in fact used FFPE samples in their study. Recently, Eliezer et al. [19] also described an approach for generating high-quality whole-exome sequencing (WES) data from archival tumor material and validated WES data obtained from FFPE tumor samples by using corresponding WES data from frozen samples. The results showed that the ability to detect base mutations with sufficient power was equivalent regardless of whether frozen or FFPE tissue-derived genomic DNA was used for WES. The power of NGS (involving genomic SNP chips) for in-depth analysis of large numbers of short sequences potentially makes this an ideal technology in the case of the fragmented nucleic acids that are usually extracted from FFPE specimens [17]. Therefore, the development of reliable NGS-based methods for use with low-quality, FFPE tissue-derived nucleic acids would enable the use of samples from diagnostic pathology archives for high-throughput profiling, thereby facilitating extensive retrospective clinical studies. Our studies showed that these two methods can be used to extract good-quality DNA for detection with the exome SNP chip (call rate, 0.7526), using FFPE SS samples.

Morphologically, the most outstanding characteristic of BSSs is the mixture of glandular solid epithelial sheets or cystic papillary epithelial components and spindle mesenchymal elements. The proportion of the epithelial component varies widely. Some biphasic components form glands with a highly atypical epithelium, and the spindle cell component is composed of uniform mildly hyperchromatic cells. The broad spectrum of histopathological and molecular sarcoma subtypes, as well as the clinical behavior of these tumors, is the reason for the delay in clinical studies and preclinical discoveries. Mertens et al. [20] reported that cytogenetic analysis has not only provided important information on the pathogenesis of soft-tissue tumors but, by identifying distinct chromosomal rearrangements in different histopathological entities, has also come to serve as a valuable diagnostic tool. Therefore, to isolate pure tumor cell populations, we demonstrate the efficacy of LCM coupled with a cored tissue chip for the isolation of DNA from epithelial and spindle components of SSs. LCM is a well-established method for the isolation of cells from biological mixtures, even when cells are found in low abundance [21]. In our previous study [22], we found significant differences between different histological subtypes and epithelial–mesenchymal composition with respect to the expression of the TGF-β1 pathway and EMT-related proteins in SS. Our functional annotation of SNP-chip data suggests that the differing morphologies of BSSs indicate different genomic changes. The differential mutations had 336 SNPs, which contained 319 genes (including TGF-β1 pathway-related genes). For example, exm1611770 in *EP300*, exm412589 in *SPP1* and exm648916 in *PIK3CG* were found to be strongly associated with BSSs. Therefore, these data perhaps support the hypothesis that neoplastic EMT contributes to the transition between the spindle cell and epithelial morphologies of SS.

Although our SNP analysis points to disease associations with many gene variants, the contribution of hereditary factors to the SS phenotypes is currently unknown. WebGestalt [6] is a suite of tools for functional enrichment analysis in various biological contexts (including GO Slim classification, directed acyclic graph [DAG] structure, KEGG pathway, WikiPathways and Pathway Commons pathway). The WebGestalt results showed that the differential SNPs in genes for functional enrichment were mainly related to the inflammatory response pathway, adhesion, ECM–receptor interactions, the TGF-β signaling pathway, the JAK–STAT signaling pathway, phenylalanine metabolism, the intrinsic pathway and fibrin formation. Moreover, those pathways that closely correlated with tight junction genes served as a signature for epithelial-like cancer cells or some invasion- and metastasis-associated genes. Kohn et al. [23] demonstrated that some highly correlated genes, including genes involved in interactions at tight junctions, adhesion junctions, desmosomes, transcription regulation of cell–cell junction complexes, epithelial vesicle traffic and epithelial Ca(+2) signaling, were implicated in epithelial functions in NCI-60 human tumor cell lines and the CCLE cell lines of the Broad Institute. Saito [24] provided a good model for this epithelial differentiation, which shows a possible mechanism for the aberrant mesenchymal-to-epithelial transition (MET) of SS and suggested that it would be better to consider it as an epithelial-to-mesenchymal transition (EMT). Therefore, these findings suggested that epithelial-like cancer cells or aberrant MET were present by analyzing the regulation of networks, whether carcinoma or sarcoma. These molecular signatures are not only important for appropriate diagnosis but also contribute to the investigation of the tumorigenic and metastatic mechanisms of cancer [25]. Our results also suggested that differing morphologies are due to different genomic changes.

## Conclusions

Clinical applications of genomic biomarkers have been rapidly expanded and developed. Here, we performed a genome-wide SNP analysis of the epithelial and spindle cell components of BSSs. We found some significant mutations in genes whose functional annotations suggested involvement in cell adhesion, ECM–ECM receptor interactions, the TGF-β signaling pathway and cell junctions and signaling. These findings closely correlate with our previous immunohistochemical studies on SS, wherein TGF-β1 was found to induce mesenchymal–epithelial transition and to regulate biphasic differentiation. Such annotations provide a framework and a new target for treatment along with strategies for continued research on the mechanism.

### Availability of supporting data

The data discussed in this publication have been deposited in LabArchives and are accessible through dataset DOI: 10.6070/H4Z31WP8 (http://www.labarchives.com/).

## Additional files

Additional file 1:
**The summary of 336 differentiated data of SNP.** (XLSX 91 kb) Additional file 2:
**Enrichment analysis of the Pathway Commons Pathway of differentiated genes.** (HTML 20 kb) Additional file 3:
**Enrichment analysis of the Wikipathways Pathway of differentiated genes.** (HTML 24 kb) Additional file 4:
**Enrichment analysis of the KEGG Pathway of differentiated genes.** (HTML 26 kb)

## Abbreviation

SS: Synovial sarcoma
BSS: Biphasic synovial sarcoma
SNP: Single nucleotide polymorphism
NGDN: Neuroguidin
RASAL3: RAS protein activator like 3
FFPE: Formalin-fixed, paraffin-embedded
LCM: Laser capture microdissection
KLHL: Kelch-like
MUM1L1: Melanoma-associated antigen (mutated) 1-like 1 gene
GO: Gene ontology
KEGG: Kyoto encyclopedia of gene and genomes
WES: Whole-exome sequencing
NGS: Next generation sequencing
EMT: Epithelial-mesenchymal transition
MET: Mesenchymal-epithelial transition
