# Supplementary material for: Identification of potential mutations and genomic alterations in the epithelial and spindle cell components of biphasic synovial sarcomas using a human exome SNP chip
Source: BMC Med Genomics. 2015 Oct 27;8:69. doi: 10.1186/s12920-015-0144-7 (PMC4621929; doi:10.1186/s12920-015-0144-7)
Supplement: Additional file 2: — Enrichment analysis of the Pathway Commons Pathway of differentiated genes. (HTML 20 kb) [file 12920_2015_144_MOESM2_ESM.html]

Anchored HTML File of EIDs


|  |  |  |  |  |  |
| --- | --- | --- | --- | --- | --- |
| **User file and parameters:** User file: genelist.txt, Organism: hsapiens, Id Type: gene\_symbol, Ref Set: illumina\_OmniExpress\_SNP, Significance Level: Top10, Statistics Test: Hypergeometric, MTC: BH, Minimum: 2  The results for each enriched gene set are listed in this table. For each gene set, the first row lists the gene set name, and corresponding Gene Set ID. The second row lists number of reference genes in the category (C), number of genes in the gene set and also in the category (O), expected number in the category (E), Ratio of enrichment (R), p value from hypergeometric test (rawP), and p value adjusted by the multiple test adjustment (adjP). Finally, genes in the pathway are listed. For each gene, the table lists the user uploaded ID and value (optional), Entrez ID, Ensembl Gene Stable ID, Gene symbol, and description. Ensembl Gene Stable ID and Entrez Gene ID are linked to the Ensembl and Entrez Gene databases, respectively. | | | | | |
| **Pathway Commons pathway----Intrinsic Pathway----DB\_ID:438** | | | | | |
| C=31;O=4;E=0.50;R=8.01;rawP=0.0015;adjP=0.1187 | | | | | |
| GP9 | NA | 2815 | ENSG00000169704 | GP9 | glycoprotein IX (platelet) |
| C1QBP | NA | 708 | ENSG00000108561 | C1QBP | complement component 1, q subcomponent binding protein |
| A2M | NA | 2 | ENSG00000175899 | A2M | alpha-2-macroglobulin |
| F13A1 | NA | 2162 | ENSG00000124491 | F13A1 | coagulation factor XIII, A1 polypeptide |
| **Pathway Commons pathway----Formation of Fibrin Clot (Clotting Cascade)----DB\_ID:870** | | | | | |
| C=34;O=4;E=0.55;R=7.30;rawP=0.0021;adjP=0.1187 | | | | | |
| GP9 | NA | 2815 | ENSG00000169704 | GP9 | glycoprotein IX (platelet) |
| C1QBP | NA | 708 | ENSG00000108561 | C1QBP | complement component 1, q subcomponent binding protein |
| A2M | NA | 2 | ENSG00000175899 | A2M | alpha-2-macroglobulin |
| F13A1 | NA | 2162 | ENSG00000124491 | F13A1 | coagulation factor XIII, A1 polypeptide |
| **Pathway Commons pathway----Hemostasis----DB\_ID:868** | | | | | |
| C=165;O=8;E=2.66;R=3.01;rawP=0.0054;adjP=0.1187 | | | | | |
| TTN | NA | 7273 | ENSG00000155657 | TTN | titin |
| COL1A1 | NA | 1277 | ENSG00000108821 | COL1A1 | collagen, type I, alpha 1 |
| A2M | NA | 2 | ENSG00000175899 | A2M | alpha-2-macroglobulin |
| EGF | NA | 1950 | ENSG00000138798 | EGF | epidermal growth factor (beta-urogastrone) |
| F13A1 | NA | 2162 | ENSG00000124491 | F13A1 | coagulation factor XIII, A1 polypeptide |
| GP9 | NA | 2815 | ENSG00000169704 | GP9 | glycoprotein IX (platelet) |
| MAG | NA | 4099 | ENSG00000105695 | MAG | myelin associated glycoprotein |
| C1QBP | NA | 708 | ENSG00000108561 | C1QBP | complement component 1, q subcomponent binding protein |
| **Pathway Commons pathway----Platelet Activation----DB\_ID:873** | | | | | |
| C=70;O=5;E=1.13;R=4.43;rawP=0.0054;adjP=0.1187 | | | | | |
| GP9 | NA | 2815 | ENSG00000169704 | GP9 | glycoprotein IX (platelet) |
| COL1A1 | NA | 1277 | ENSG00000108821 | COL1A1 | collagen, type I, alpha 1 |
| A2M | NA | 2 | ENSG00000175899 | A2M | alpha-2-macroglobulin |
| EGF | NA | 1950 | ENSG00000138798 | EGF | epidermal growth factor (beta-urogastrone) |
| F13A1 | NA | 2162 | ENSG00000124491 | F13A1 | coagulation factor XIII, A1 polypeptide |
| **Pathway Commons pathway----Exocytosis of Alpha granule----DB\_ID:397** | | | | | |
| C=43;O=4;E=0.69;R=5.78;rawP=0.0050;adjP=0.1187 | | | | | |
| GP9 | NA | 2815 | ENSG00000169704 | GP9 | glycoprotein IX (platelet) |
| A2M | NA | 2 | ENSG00000175899 | A2M | alpha-2-macroglobulin |
| EGF | NA | 1950 | ENSG00000138798 | EGF | epidermal growth factor (beta-urogastrone) |
| F13A1 | NA | 2162 | ENSG00000124491 | F13A1 | coagulation factor XIII, A1 polypeptide |
| **Pathway Commons pathway----Platelet degranulation----DB\_ID:396** | | | | | |
| C=45;O=4;E=0.72;R=5.52;rawP=0.0059;adjP=0.1187 | | | | | |
| GP9 | NA | 2815 | ENSG00000169704 | GP9 | glycoprotein IX (platelet) |
| A2M | NA | 2 | ENSG00000175899 | A2M | alpha-2-macroglobulin |
| EGF | NA | 1950 | ENSG00000138798 | EGF | epidermal growth factor (beta-urogastrone) |
| F13A1 | NA | 2162 | ENSG00000124491 | F13A1 | coagulation factor XIII, A1 polypeptide |
| **Pathway Commons pathway----Terminal pathway of complement----DB\_ID:165** | | | | | |
| C=6;O=2;E=0.10;R=20.70;rawP=0.0037;adjP=0.1187 | | | | | |
| C8G | NA | 733 | ENSG00000176919 | C8G | complement component 8, gamma polypeptide |
| C7 | NA | 730 | ENSG00000112936 | C7 | complement component 7 |
| **Pathway Commons pathway----vWF interaction with collagen----DB\_ID:332** | | | | | |
| C=6;O=2;E=0.10;R=20.70;rawP=0.0037;adjP=0.1187 | | | | | |
| GP9 | NA | 2815 | ENSG00000169704 | GP9 | glycoprotein IX (platelet) |
| COL1A1 | NA | 1277 | ENSG00000108821 | COL1A1 | collagen, type I, alpha 1 |
| **Pathway Commons pathway----Activation of C3 and C5----DB\_ID:504** | | | | | |
| C=9;O=2;E=0.14;R=13.80;rawP=0.0086;adjP=0.1385 | | | | | |
| C8G | NA | 733 | ENSG00000176919 | C8G | complement component 8, gamma polypeptide |
| C7 | NA | 730 | ENSG00000112936 | C7 | complement component 7 |
| **Pathway Commons pathway----Platelet Adhesion to exposed collagen----DB\_ID:713** | | | | | |
| C=9;O=2;E=0.14;R=13.80;rawP=0.0086;adjP=0.1385 | | | | | |
| GP9 | NA | 2815 | ENSG00000169704 | GP9 | glycoprotein IX (platelet) |
| COL1A1 | NA | 1277 | ENSG00000108821 | COL1A1 | collagen, type I, alpha 1 |
