# Supplementary material for: Identification of potential mutations and genomic alterations in the epithelial and spindle cell components of biphasic synovial sarcomas using a human exome SNP chip
Source: BMC Med Genomics. 2015 Oct 27;8:69. doi: 10.1186/s12920-015-0144-7 (PMC4621929; doi:10.1186/s12920-015-0144-7)
Supplement: Additional file 3: — Enrichment analysis of the Wikipathways Pathway of differentiated genes. (HTML 24 kb) [file 12920_2015_144_MOESM3_ESM.html]

Anchored HTML File of EIDs


|  |  |  |  |  |  |
| --- | --- | --- | --- | --- | --- |
| **User file and parameters:** User file: genelist.txt, Organism: hsapiens, Id Type: gene\_symbol, Ref Set: illumina\_OmniExpress\_SNP, Significance Level: Top10, Statistics Test: Hypergeometric, MTC: BH, Minimum: 2  The results for each enriched gene set are listed in this table. For each gene set, the first row lists the gene set name, and corresponding Gene Set ID. The second row lists number of reference genes in the category (C), number of genes in the gene set and also in the category (O), expected number in the category (E), Ratio of enrichment (R), p value from hypergeometric test (rawP), and p value adjusted by the multiple test adjustment (adjP). Finally, genes in the pathway are listed. For each gene, the table lists the user uploaded ID and value (optional), Entrez ID, Ensembl Gene Stable ID, Gene symbol, and description. Ensembl Gene Stable ID and Entrez Gene ID are linked to the Ensembl and Entrez Gene databases, respectively. | | | | | |
| **Wikipathways pathway----Inflammatory Response Pathway----WP453** | | | | | |
| C=30;O=4;E=0.48;R=8.28;rawP=0.0013;adjP=0.0481 | | | | | |
| IL4R | NA | 3566 | ENSG00000077238 | IL4R | interleukin 4 receptor |
| LAMC2 | NA | 3918 | ENSG00000058085 | LAMC2 | laminin, gamma 2 |
| LAMA5 | NA | 3911 | ENSG00000130702 | LAMA5 | laminin, alpha 5 |
| COL1A1 | NA | 1277 | ENSG00000108821 | COL1A1 | collagen, type I, alpha 1 |
| **Wikipathways pathway----Focal Adhesion----WP306** | | | | | |
| C=183;O=9;E=2.95;R=3.05;rawP=0.0029;adjP=0.0537 | | | | | |
| SPP1 | NA | 6696 | ENSG00000118785 | SPP1 | secreted phosphoprotein 1 |
| COL1A1 | NA | 1277 | ENSG00000108821 | COL1A1 | collagen, type I, alpha 1 |
| COL5A1 | NA | 1289 | ENSG00000130635 | COL5A1 | collagen, type V, alpha 1 |
| EGF | NA | 1950 | ENSG00000138798 | EGF | epidermal growth factor (beta-urogastrone) |
| TNXB | NA | 7148 | ENSG00000168477 | TNXB | tenascin XB |
| ROCK1 | NA | 6093 | ENSG00000067900 | ROCK1 | Rho-associated, coiled-coil containing protein kinase 1 |
| LAMC2 | NA | 3918 | ENSG00000058085 | LAMC2 | laminin, gamma 2 |
| PIK3CG | NA | 5294 | ENSG00000105851 | PIK3CG | phosphoinositide-3-kinase, catalytic, gamma polypeptide |
| LAMA5 | NA | 3911 | ENSG00000130702 | LAMA5 | laminin, alpha 5 |
| **Wikipathways pathway----TGF Beta Signaling Pathway----WP560** | | | | | |
| C=52;O=4;E=0.84;R=4.78;rawP=0.0097;adjP=0.1196 | | | | | |
| EP300 | NA | 2033 | ENSG00000100393 | EP300 | E1A binding protein p300 |
| SPP1 | NA | 6696 | ENSG00000118785 | SPP1 | secreted phosphoprotein 1 |
| JAK1 | NA | 3716 | ENSG00000162434 | JAK1 | Janus kinase 1 |
| EGF | NA | 1950 | ENSG00000138798 | EGF | epidermal growth factor (beta-urogastrone) |
| **Wikipathways pathway----Nuclear Receptors----WP170** | | | | | |
| C=38;O=3;E=0.61;R=4.90;rawP=0.0230;adjP=0.1332 | | | | | |
| ROR1 | NA | 4919 | ENSG00000185483 | ROR1 | receptor tyrosine kinase-like orphan receptor 1 |
| ESR1 | NA | 2099 | ENSG00000091831 | ESR1 | estrogen receptor 1 |
| NR5A1 | NA | 2516 | ENSG00000136931 | NR5A1 | nuclear receptor subfamily 5, group A, member 1 |
| **Wikipathways pathway----Striated Muscle Contraction----WP383** | | | | | |
| C=36;O=3;E=0.58;R=5.17;rawP=0.0200;adjP=0.1332 | | | | | |
| TMOD1 | NA | 7111 | ENSG00000136842 | TMOD1 | tropomodulin 1 |
| TTN | NA | 7273 | ENSG00000155657 | TTN | titin |
| NEB | NA | 4703 | ENSG00000183091 | NEB | nebulin |
| **Wikipathways pathway----Alpha6-Beta4 Integrin Signaling Pathway----WP244** | | | | | |
| C=69;O=4;E=1.11;R=3.60;rawP=0.0252;adjP=0.1332 | | | | | |
| DST | NA | 667 | ENSG00000151914 | DST | dystonin |
| LAMC2 | NA | 3918 | ENSG00000058085 | LAMC2 | laminin, gamma 2 |
| LAMA5 | NA | 3911 | ENSG00000130702 | LAMA5 | laminin, alpha 5 |
| PIK3CG | NA | 5294 | ENSG00000105851 | PIK3CG | phosphoinositide-3-kinase, catalytic, gamma polypeptide |
| **Wikipathways pathway----Regulation of Actin Cytoskeleton----WP51** | | | | | |
| C=134;O=6;E=2.16;R=2.78;rawP=0.0215;adjP=0.1332 | | | | | |
| ROCK1 | NA | 6093 | ENSG00000067900 | ROCK1 | Rho-associated, coiled-coil containing protein kinase 1 |
| FGFR2 | NA | 2263 | ENSG00000066468 | FGFR2 | fibroblast growth factor receptor 2 |
| PIK3CG | NA | 5294 | ENSG00000105851 | PIK3CG | phosphoinositide-3-kinase, catalytic, gamma polypeptide |
| PIK3C2A | NA | 5286 | ENSG00000011405 | PIK3C2A | phosphoinositide-3-kinase, class 2, alpha polypeptide |
| EGF | NA | 1950 | ENSG00000138798 | EGF | epidermal growth factor (beta-urogastrone) |
| FGD1 | NA | 2245 | ENSG00000102302 | FGD1 | FYVE, RhoGEF and PH domain containing 1 |
| **Wikipathways pathway----Complement and Coagulation Cascades KEGG----WP558** | | | | | |
| C=49;O=3;E=0.79;R=3.80;rawP=0.0443;adjP=0.2049 | | | | | |
| C8G | NA | 733 | ENSG00000176919 | C8G | complement component 8, gamma polypeptide |
| C7 | NA | 730 | ENSG00000112936 | C7 | complement component 7 |
| A2M | NA | 2 | ENSG00000175899 | A2M | alpha-2-macroglobulin |
| **Wikipathways pathway----Signal Transduction of S1P Receptor----WP26** | | | | | |
| C=24;O=2;E=0.39;R=5.17;rawP=0.0566;adjP=0.2327 | | | | | |
| PIK3C2B | NA | 5287 | ENSG00000133056 | PIK3C2B | phosphoinositide-3-kinase, class 2, beta polypeptide |
| RACGAP1 | NA | 29127 | ENSG00000161800 | RACGAP1 | Rac GTPase activating protein 1 |
| **Wikipathways pathway----EGFR1 Signaling Pathway----WP437** | | | | | |
| C=174;O=6;E=2.80;R=2.14;rawP=0.0629;adjP=0.2327 | | | | | |
| PIK3C2B | NA | 5287 | ENSG00000133056 | PIK3C2B | phosphoinositide-3-kinase, class 2, beta polypeptide |
| JAK1 | NA | 3716 | ENSG00000162434 | JAK1 | Janus kinase 1 |
| PIK3CG | NA | 5294 | ENSG00000105851 | PIK3CG | phosphoinositide-3-kinase, catalytic, gamma polypeptide |
| EGF | NA | 1950 | ENSG00000138798 | EGF | epidermal growth factor (beta-urogastrone) |
| EPS8 | NA | 2059 | ENSG00000151491 | EPS8 | epidermal growth factor receptor pathway substrate 8 |
| CBLB | NA | 868 | ENSG00000114423 | CBLB | Cas-Br-M (murine) ecotropic retroviral transforming sequence b |
