# Supplementary material for: Identification of potential mutations and genomic alterations in the epithelial and spindle cell components of biphasic synovial sarcomas using a human exome SNP chip
Source: BMC Med Genomics. 2015 Oct 27;8:69. doi: 10.1186/s12920-015-0144-7 (PMC4621929; doi:10.1186/s12920-015-0144-7)
Supplement: Additional file 4: — Enrichment analysis of the KEGG Pathway of differentiated genes. (HTML 26 kb) [file 12920_2015_144_MOESM4_ESM.html]

Anchored HTML File of EIDs


|  |  |  |  |  |  |
| --- | --- | --- | --- | --- | --- |
| **User file and parameters:** User file: genelist.txt, Organism: hsapiens, Id Type: gene\_symbol, Ref Set: illumina\_OmniExpress\_SNP, Significance Level: Top10, Statistics Test: Hypergeometric, MTC: BH, Minimum: 2  The results for each enriched KEGG pathway are listed in this table. For each KEGG pathway, the first row lists the KEGG pathway name, and corresponding KEGG ID. The second row lists number of reference genes in the category (C), number of genes in the gene set and also in the category (O), expected number in the category (E), Ratio of enrichment (R), p value from hypergeometric test (rawP), and p value adjusted by the multiple test adjustment (adjP). Finally, genes in the pathway are listed. For each gene, the table lists the user uploaded ID and value (optional), Entrez ID, Ensembl Gene Stable ID, Gene symbol, and description. Ensembl Gene Stable ID and Entrez Gene ID are linked to the Ensembl and Entrez Gene databases, respectively. | | | | | |
| **KEGG pathway----ECM-receptor interaction----04512** | | | | | |
| C=83;O=7;E=1.34;R=5.24;rawP=0.0004;adjP=0.0276 | | | | | |
| GP9 | NA | 2815 | ENSG00000169704 | GP9 | glycoprotein IX (platelet) |
| SPP1 | NA | 6696 | ENSG00000118785 | SPP1 | secreted phosphoprotein 1 |
| LAMA5 | NA | 3911 | ENSG00000130702 | LAMA5 | laminin, alpha 5 |
| LAMC2 | NA | 3918 | ENSG00000058085 | LAMC2 | laminin, gamma 2 |
| COL1A1 | NA | 1277 | ENSG00000108821 | COL1A1 | collagen, type I, alpha 1 |
| COL5A1 | NA | 1289 | ENSG00000130635 | COL5A1 | collagen, type V, alpha 1 |
| TNXB | NA | 7148 | ENSG00000168477 | TNXB | tenascin XB |
| **KEGG pathway----Focal adhesion----04510** | | | | | |
| C=199;O=10;E=3.21;R=3.12;rawP=0.0015;adjP=0.0517 | | | | | |
| SPP1 | NA | 6696 | ENSG00000118785 | SPP1 | secreted phosphoprotein 1 |
| FLT4 | NA | 2324 | ENSG00000037280 | FLT4 | fms-related tyrosine kinase 4 |
| COL1A1 | NA | 1277 | ENSG00000108821 | COL1A1 | collagen, type I, alpha 1 |
| COL5A1 | NA | 1289 | ENSG00000130635 | COL5A1 | collagen, type V, alpha 1 |
| EGF | NA | 1950 | ENSG00000138798 | EGF | epidermal growth factor (beta-urogastrone) |
| TNXB | NA | 7148 | ENSG00000168477 | TNXB | tenascin XB |
| ROCK1 | NA | 6093 | ENSG00000067900 | ROCK1 | Rho-associated, coiled-coil containing protein kinase 1 |
| LAMC2 | NA | 3918 | ENSG00000058085 | LAMC2 | laminin, gamma 2 |
| LAMA5 | NA | 3911 | ENSG00000130702 | LAMA5 | laminin, alpha 5 |
| PIK3CG | NA | 5294 | ENSG00000105851 | PIK3CG | phosphoinositide-3-kinase, catalytic, gamma polypeptide |
| **KEGG pathway----Phenylalanine metabolism----00360** | | | | | |
| C=21;O=3;E=0.34;R=8.87;rawP=0.0044;adjP=0.1012 | | | | | |
| IL4I1 | NA | 259307 | ENSG00000104951 | IL4I1 | interleukin 4 induced 1 |
| AOC2 | NA | 314 | ENSG00000131480 | AOC2 | amine oxidase, copper containing 2 (retina-specific) |
| ALDH3B2 | NA | 222 | ENSG00000132746 | ALDH3B2 | aldehyde dehydrogenase 3 family, member B2 |
| **KEGG pathway----Jak-STAT signaling pathway----04630** | | | | | |
| C=135;O=7;E=2.17;R=3.22;rawP=0.0063;adjP=0.1087 | | | | | |
| IFNE | NA | 338376 | NULL | IFNE | interferon, epsilon |
| EP300 | NA | 2033 | ENSG00000100393 | EP300 | E1A binding protein p300 |
| IL4R | NA | 3566 | ENSG00000077238 | IL4R | interleukin 4 receptor |
| IL11RA | NA | 3590 | ENSG00000137070 | IL11RA | interleukin 11 receptor, alpha |
| JAK1 | NA | 3716 | ENSG00000162434 | JAK1 | Janus kinase 1 |
| PIK3CG | NA | 5294 | ENSG00000105851 | PIK3CG | phosphoinositide-3-kinase, catalytic, gamma polypeptide |
| CBLB | NA | 868 | ENSG00000114423 | CBLB | Cas-Br-M (murine) ecotropic retroviral transforming sequence b |
| **KEGG pathway----Hematopoietic cell lineage----04640** | | | | | |
| C=78;O=5;E=1.26;R=3.98;rawP=0.0085;adjP=0.1173 | | | | | |
| CD1E | NA | 913 | ENSG00000158488 | CD1E | CD1e molecule |
| GP9 | NA | 2815 | ENSG00000169704 | GP9 | glycoprotein IX (platelet) |
| IL4R | NA | 3566 | ENSG00000077238 | IL4R | interleukin 4 receptor |
| IL11RA | NA | 3590 | ENSG00000137070 | IL11RA | interleukin 11 receptor, alpha |
| CD22 | NA | 933 | ENSG00000012124 | CD22 | CD22 molecule |
| **KEGG pathway----Inositol phosphate metabolism----00562** | | | | | |
| C=54;O=4;E=0.87;R=4.60;rawP=0.0111;adjP=0.1277 | | | | | |
| PLCB4 | NA | 5332 | ENSG00000101333 | PLCB4 | phospholipase C, beta 4 |
| PIK3C2B | NA | 5287 | ENSG00000133056 | PIK3C2B | phosphoinositide-3-kinase, class 2, beta polypeptide |
| PIK3CG | NA | 5294 | ENSG00000105851 | PIK3CG | phosphoinositide-3-kinase, catalytic, gamma polypeptide |
| PIK3C2A | NA | 5286 | ENSG00000011405 | PIK3C2A | phosphoinositide-3-kinase, class 2, alpha polypeptide |
| **KEGG pathway----Complement and coagulation cascades----04610** | | | | | |
| C=68;O=4;E=1.10;R=3.65;rawP=0.0240;adjP=0.2239 | | | | | |
| C8G | NA | 733 | ENSG00000176919 | C8G | complement component 8, gamma polypeptide |
| C7 | NA | 730 | ENSG00000112936 | C7 | complement component 7 |
| A2M | NA | 2 | ENSG00000175899 | A2M | alpha-2-macroglobulin |
| F13A1 | NA | 2162 | ENSG00000124491 | F13A1 | coagulation factor XIII, A1 polypeptide |
| **KEGG pathway----Phosphatidylinositol signaling system----04070** | | | | | |
| C=76;O=4;E=1.22;R=3.27;rawP=0.0343;adjP=0.2239 | | | | | |
| PLCB4 | NA | 5332 | ENSG00000101333 | PLCB4 | phospholipase C, beta 4 |
| PIK3C2B | NA | 5287 | ENSG00000133056 | PIK3C2B | phosphoinositide-3-kinase, class 2, beta polypeptide |
| PIK3CG | NA | 5294 | ENSG00000105851 | PIK3CG | phosphoinositide-3-kinase, catalytic, gamma polypeptide |
| PIK3C2A | NA | 5286 | ENSG00000011405 | PIK3C2A | phosphoinositide-3-kinase, class 2, alpha polypeptide |
| **KEGG pathway----ABC transporters----02010** | | | | | |
| C=44;O=3;E=0.71;R=4.23;rawP=0.0337;adjP=0.2239 | | | | | |
| ABCC2 | NA | 1244 | ENSG00000023839 | ABCC2 | ATP-binding cassette, sub-family C (CFTR/MRP), member 2 |
| ABCB1 | NA | 5243 | ENSG00000085563 | ABCB1 | ATP-binding cassette, sub-family B (MDR/TAP), member 1 |
| ABCA8 | NA | 10351 | ENSG00000141338 | ABCA8 | ATP-binding cassette, sub-family A (ABC1), member 8 |
| **KEGG pathway----Tyrosine metabolism----00350** | | | | | |
| C=45;O=3;E=0.72;R=4.14;rawP=0.0357;adjP=0.2239 | | | | | |
| IL4I1 | NA | 259307 | ENSG00000104951 | IL4I1 | interleukin 4 induced 1 |
| AOC2 | NA | 314 | ENSG00000131480 | AOC2 | amine oxidase, copper containing 2 (retina-specific) |
| ALDH3B2 | NA | 222 | ENSG00000132746 | ALDH3B2 | aldehyde dehydrogenase 3 family, member B2 |
